# NTS/NTR1 co-expression enhances epithelial-to-mesenchymal transition and promotes tumor metastasis by activating the Wnt/β-catenin signaling pathway in hepatocellular carcinoma

**DOI:** 10.18632/oncotarget.11854

**Published:** 2016-09-06

**Authors:** Yingnan Ye, Xinxin Long, Lijie Zhang, Jieying Chen, Pengpeng Liu, Hui Li, Feng Wei, Wenwen Yu, Xiubao Ren, Jinpu Yu

**Affiliations:** ^1^ Cancer Molecular Diagnostic Center, Tianjin Medical University Cancer Institute & Hospital, National Clinical Research Center of Caner, Key Laboratory of Cancer Prevention and Therapy, Tianjin, P. R. China; ^2^ Department of Immunology, Tianjin Medical University Cancer Institute & Hospital, National Clinical Research Center of Caner, Key Laboratory of Cancer Immunology and Biotherapy, Tianjin, P. R. China; ^3^ Department of Gastrointestinal Cancer Biology, Tianjin Medical University Cancer Institute & Hospital, National Clinical Research Center of Caner, Key Laboratory of Cancer Prevention and Therapy, Tianjin, P. R. China

**Keywords:** neurotensin, hepatocellular carcinoma, epithelial–mesenchymal transition, Wnt/β-catenin

## Abstract

Neurotensin (NTS) is a neuropeptide distributed in central nervous and digestive systems. In this study, the significant association between ectopic NTS expression and tumor invasion was confirmed in hepatocellular carcinoma (HCC). In primary HCC tissues, the NTS and neurotensin receptor 1 (NTR1) co-expression (NTS^+^NTR1^+^) is a poor prognostic factor correlated with aggressive biological behaviors and poor clinical prognosis. Enhanced epithelial-to-mesenchymal transition (EMT) features, including decreased E-cadherin, increased β-catenin translocation and N-cadherin expression, were identified in NTS^+^NTR1^+^ HCC tissues. Varied NTS-responsible HCC cell lines were established using NTR1 genetically modified Hep3B and HepG2 cells which were used to elucidate the molecular mechanisms regulating NTS-induced EMT and tumor invasion *in vitro*. Results revealed that inducing exogenous NTS stimulation and enhancing NTR1 expression promoted tumor invasion rather than proliferation by accelerating EMT in HCC cells. The NTS-induced EMT was correlated with the remarkable increase in Wnt1, Wnt3, Wnt5, Axin, and p-GSK3β expression and was significantly reversed by blocking the NTS signaling via the NTR1 antagonist SR48692 or by inhibiting the activation of the Wnt/β-catenin pathway via specific inhibitors, such as TSW119 and DKK-1. SR48692 also inhibited the metastases of NTR1-overexpressing HCC xenografts in the lungs *in vivo*. This finding implied that NTS may be an important stimulus to promote HCC invasion and metastasis both *in vitro* and *in vivo*, and NTS signaling enhanced the tumor EMT and invasion potentials by activating the canonical Wnt/β-catenin signaling pathway. Therefore, NTS may be a valuable therapeutic target to prevent tumor progression in HCC.

## INTRODUCTION

Hepatocellular carcinoma (HCC) is the seventh-most common malignancy and third leading cause of cancer-related deaths worldwide [1]. Epithelial-to-mesenchymal transition (EMT) plays a crucial role in the dissemination and invasion of HCC, which significantly affects clinical prognosis. Growth factors and cytokine-induced signal pathways, such as Wnt/β-catenin, TGF-β/SMAD, JAK2/STAT3, and NF-κB pathways, participate in the EMT of HCC cells [2–5]. Recent study in prostate cancer indicated that the acquisition of EMT features and the preservation of cancer stem cell phenotype are associated with the neuroendocrine system, which implied that neuropeptides might facilitate tumor evasion and distant metastasis by promoting the development and maintenance of EMT in cancer cells [6]. However, whether neuropeptides influences the tumor invasion and EMT of HCC remains unknown.

Neurotensin (NTS) is an endogenous neuropeptide with 13 amino acids and was first isolated from the extracts of bovine hypothalami in 1973 [7]. In the periphery, NTS is released from entero-endocrine N cells of the gastrointestinal tract and involved in digestion-related hormonal and neurocrine regulatory processes, such as inhibiting small bowel motility and gastric acid secretions, stimulating pancreatic and biliary secretions, and facilitating fatty acid absorption [8, 9]. The ectopic expression of NTS was first identified in breast cancer in 1985 [10]. Since then, the expression of NTS has been detected in pancreatic, colon, prostate and lung cancers and neuroendocrine tumors [11–13]. In our previous study, the mRNA expression level of NTS is comparably increased in a special HCC subgroup characterized by an enhanced expression of inflammatory and EMT-related genes, as revealed by a comprehensive genome-wide gene expression profiling analysis of 41 cases of Asian HCC tissues [14]. In primary HCC tissues, the NTS protein is positively expressed and a series of EMT biomarkers is upregulated, as indicated by immunohistochemistry (IHC) staining [14]. These findings implied that NTS might promote tumor EMT in HCC.

Previous studies on the role of NTS in cancer focused on tumor growth, proliferation, and anti-apoptosis. The effects of NTS are mediated by three subtypes of neurotensin receptors (NTRs), namely, NTR1, NTR2, and SORT1; among these receptors, NTR1 exhibits the highest affinity and mediates most biological functions of NTS [15]. NTS stimulates the proliferation of NTR1-expressing human colon cancer cell lines [16]. NTR1 knockdown or NTR1 antagonist treatment reduces the proliferation of lung cancer cell line A549 *in vitro* and *in vivo* [17]. NTS enhances the proliferation of Panc-1 cell line, and the NTS antagonist SR48692 inhibits its growth in a dose-dependent manner [18]. The anti-apoptotic effects of NTS have also been detected in breast cancer cell lines and primary tumor tissues [11, 19]. NTS also participates in tumor migration and invasion. In prostate cancer, NTS stimulates tumor invasion rather than growth [20, 21]. However, the expression of NTS in HCC has been rarely explored because NTS is absent in healthy adult liver. Therefore, we investigated the expression and biological functions of NTS in tumor proliferation, apoptosis, invasion, and metastasis in HCC tissues, gene-modified HCC cell lines, and HCC xenograft-bearing mouse models to elucidate the regulatory effects of NTS signaling in HCC.

We found that NTS and NTR1 co-expression (NTS^+^NTR1^+^) was correlated with the aggressive HCC phenotypes, including incomplete envelope, portal vein invasion, early relapse, and short survival after surgery. Significantly enhanced EMT features, such as decreased E-cadherin, increased β-catenin translocation, and increased N-cadherin expression, were also identified in NTS^+^NTR1^+^ HCC samples. Exogenous NTS stimulation and NTR1 expression enhancement promoted tumor migration and invasion rather than proliferation and apoptosis *in vitro*. In addition to the increase in EMT markers, remarkable upregulation of Wnt1, Wnt3, Wnt5, Axin, and p-GSK3β were observed. The specific inhibitors against NTR1, Wnt, and GSK3β potently blocked the transduction of NTS signal and inhibited the activation of the Wnt/β-catenin pathway. Thus, EMT was reversed and the HCC invasion capacity was attenuated *in vitro* and *in vivo*. Our study indicated that NTS signaling enhanced the tumor EMT and invasion potentials by activating the canonical Wnt/β-catenin signaling pathway in HCC. Therefore, NTS may be an important stimulus to promote HCC metastasis and valuable therapeutic target to prevent tumor progression.

## RESULTS

### NTS/NTR1 co-expression was correlated with aggressive phenotypes of HCC and poor clinical outcome

**Figure 1:**
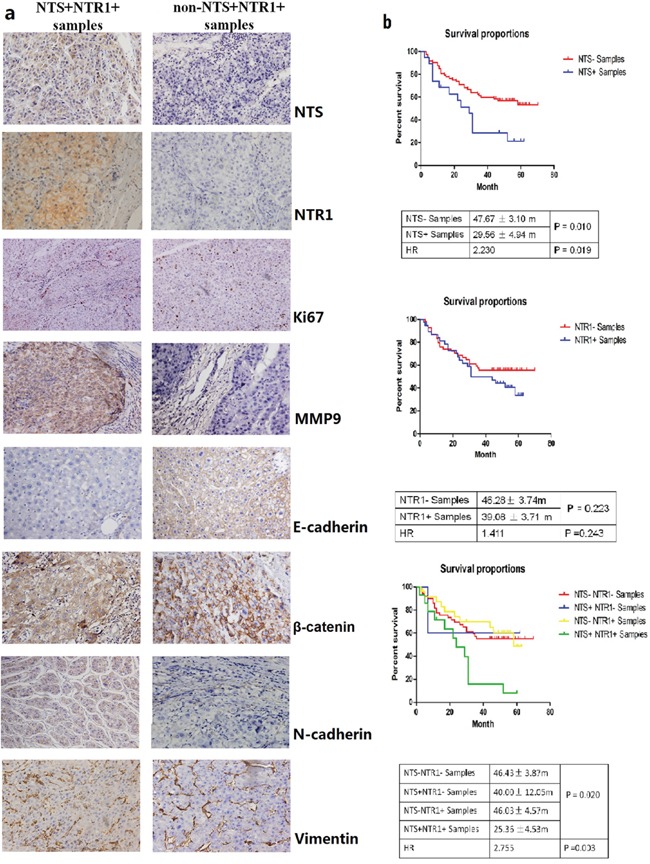
NTS/NTR1 co-expression was correlated with aggressive phenotype and poor clinical outcome of HCC **a.** NTS and NTR1 were exclusively expressed in HCC cancer cells rather than normal adjacent tissues. The proliferation marker Ki67 and the metastasis marker MMP9 as well as some EMT-related proteins, such as E-cadherin, N-cadherin, β-catenin, and Vimentin, were compared between NTS^+^NTR1^+^ and non-NTS^+^NTR1^+^ samples using IHC. Significant upregulation of MMP9 and multiple EMT markers, but not Ki67 protein was found in NTS^+^NTR1^+^ samples compared with those in non-NTS^+^NTR1^+^ samples. Decreased expression of E-cadherin and increased expression of N-cadherin on the cytomembrane and accumulation of β-catenin in the cytoplasm were found in the NTS^+^NTR1^+^ samples. No significant difference of Vimentin was identified. **b.** The survival analysis of HCC patients on the basis of the NTS and NTR1 expression was performed using Kaplan-Meier method and log-rank test. NTS exerted a significant influence on the OS of HCC patients, in which NTS^+^ patients suffered from shorter OS than NTS^−^ patients did, but no significant difference of OS was observed between NTR1^+^ and NTR1^−^ patients. NTS^+^NTR1^+^ patients exhibited a shorter OS of 25.36 ± 4.53 m (P = 0.020) and a higher death risk at an expected hazard ratio (HR) of 2.755 (P = 0.003) than the other patients did.

The expression levels of NTS and NTR1 in HCC were assessed in 100 cases of primary HCC tissues through IHC staining. Among the HCC samples, the frequencies of NTS-expressing (NTS^+^) and NTR1-expressing (NTR1^+^) tissues were 19.00% (19/100) and 41.00% (41/100), respectively. HCC cells exclusively expressed and released NTS in tumor tissues, and most of NTS-positive HCC tissues (73.68%) co-expressed NTR1. NTS-positive staining signals were exclusively detected in the cytoplasm of HCC cells. NTR1 was also observed in the cytoplasm and on the membrane of HCC cells. No positive staining of both molecules was observed in the normal adjacent tissues (Figure 1a). The average positive rate (PR) of the NTS^+^ samples was 47.3% (32.5%, 63.1%) and their average staining index (SI) was 2.65 ± 0.75. By comparison, the average PR of the NTR1^+^ samples was 25.0% (13.5%, 58.2%), and the average SI was 2.43 ± 0.68. This finding implied that the expression of NTS and NTR1 in HCC tissues was considerably strong, although the positive frequency was low (Figure 1b). Considering the correlation between multiple clinical–pathological features and both molecules in HCC tissues, we found that NTS^+^NTR1^+^ was significantly correlated with hepatic envelope completion and portal vein invasion (Table 1). We also compared the overall survival (OS) of HCC patients on the basis of the NTS and NTR1 expression. Kaplan–Meier survival analysis revealed that NTS significantly influenced the OS of HCC patients. NTS^+^ patients suffered from shorter OS than NTS^−^ patients did (29.56 ± 4.94 m vs. 47.67 ± 3.10 m, P = 0.010).However, the OS of NTR1^+^ and NTR1^−^ patients did not significantly differ (39.08 ± 3.71 m vs. 46.28 ± 3.74 m, P = 0.223; Figure 1b). NTS^+^NTR1^+^ patients exhibited a shorter OS of 25.36 ± 4.53 m (P = 0.020) and a higher death risk at an expected hazard ratio (HR) of 2.755 (P = 0.003) than the other patients did (Figure 1b). This finding implied that NTS^+^NTR1^+^ is a poor prognostic factor of HCC correlated with aggressive biological behavior and poor clinical prognosis.

**Table 1:** The correlation between NTS and/or NTR1 high expression in HCC tissues and multiple clinical-pathological features of patients

Clinicopathological parameters	n	NTS		P value
		NTS	NTS	
**Age (year)**				
<60	68	0.256	0.093	0.539
≥60	32			
**Gender**				
Male	93	0.095	0.523	0.589
Female	7			
**HBV infection**				
Positive	79	0.536	0.012	0.728
Negative	21			
**Alcohol**				
Yes	33	0.139	0.286	0.112
No	67			
**Differentiation**				
Well	26	0.423	0.081	0.371
Moderately	36			
Poorly	38			
**Tumor size**				
<5cm	59	0.531	0.319	0.879
≥5cm	41			
**Envelope**				
Yes	39	0.005	0.001	0.028
No	61			
**Portal vein invasion**				
Yes	42	0.001	0.001	0.016
No	58			

Tumor proliferation- and invasion-related biomarkers, including Ki67, MMP9, E-cadherin, N-cadherin, β-catenin, and Vimentin, were examined in 100 HCC primary tissue samples to determine whether NTS^+^NTR1^+^ promotes tumor invasion. MMP9 and multiple EMT markers, but not Ki67 protein, were significantly upregulated in NTS^+^NTR1^+^ samples compared with those in non-NTS^+^NTR1^+^ samples (P <0.001, Figure 1a). The expression of E-cadherin remarkably decreased, and its average PR in the NTS^+^NTR1^+^ samples was lower than that in the non-NTS^+^NTR1^+^ samples [0(0, 15.25)% vs. 15.33(5.25, 37.5)%;P = 0.027]. N-cadherin on the cytomembrane and β-catenin in the cytoplasm exhibited comparable increases in the NTS^+^NTR1^+^samples compared with those in the non-NTS^+^NTR1^+^ samples [25.85(7.15, 53.75)% vs. 10.25(0, 18.5)%; P = 0.039 and 20.85(7.15, 55.45)% vs. 10.0(0, 20.65)%; P = 0.029]. Furthermore, the NTS^+^NTR1^+^was significantly associated with MMP9, E-cadherin, N-cadherin, and β-catenin (P = 0.001, 0.033, 0.041, and 0.004). These results indicated that the ectopic NTS^+^NTR1^+^ promoted tumor invasion and metastasis by inducing EMT in HCC cells.

### NTS/NTR1 co-expression was correlated with tumor invasion potentials of HCC cell lines

**Figure 2:**
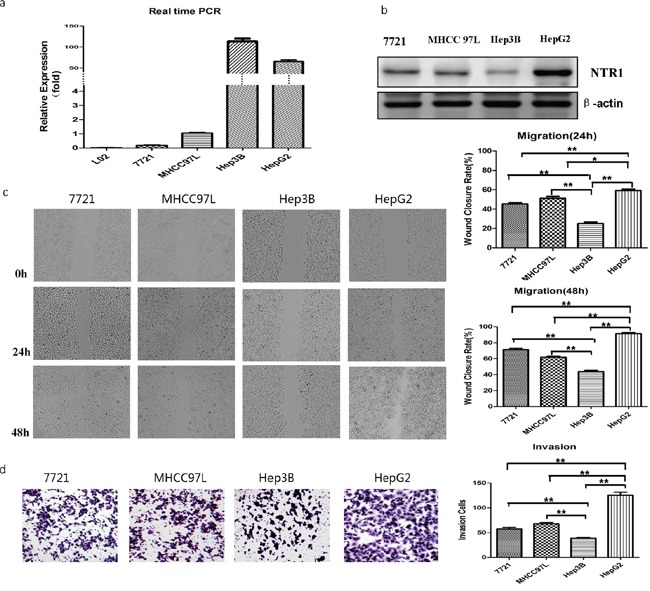
NTS/NTR1 co-expression was correlated with tumor invasion potentials of HCC cell lines **a.** The expression level of NTS was detected using real-time RT-PCR assay. NTS was absent in the normal liver cell L02, but was expressed exclusively in the HCC cells. In these cells, Hep3B and HepG2 produced more NTS than the others did. **b.** The expression level of NTR1 was detected using Western blot. HepG2 cells expressed a higher level of NTR1 protein than the Hep3B cells did, indicating that stronger NTS signal in HepG2 cells than in Hep3B cells. **c.** The migration capacity of 4 HCC cell lines was analyzed using wound healing test, and HepG2 cells displayed a higher migration capacity than Hep3B cells did. **d.** The invasion potential of 4 HCC cell lines was evaluated through Transwell invasion assay, and HepG2 cells displayed higher invasion potentials than Hep3B cells did.

NTS was transiently expressed in embryonic liver and was not expressed in normal mature liver [22]. Our results demonstrated that NTS was expressed exclusively in HCC cells and not in normal adjacent tissues. Therefore, the expression levels of NTS and NTR1 in the normal liver cell line L02 and four HCC cell lines (7721, MHCC97L, Hep3B, and HepG2) were compared at RNA or protein levels. NTS was absent in the normal liver cell L02 but was expressed exclusively in the HCC cells; in these cells, Hep3B and HepG2 produced more NTS than the others did (Figure 2a; P < 0.01). We then examined the expression of the high-affinity receptor NTR1 in the four HCC cell lines. HepG2 cells expressed a higher level of NTR1 protein than the Hep3B cells did. This finding suggested that the NTS signal was stronger in HepG2 cells than in Hep3B cells (Figure 2b; P < 0.01). The migration and invasion of four HCC cell lines were also compared through wound healing test and Transwell invasion assay. Migration and invasion significantly differed among the four HCC cell lines. HepG2 cells displayed a higher migration capacity (Figure 2c; P < 0.01) and invasion potentials (Figure 2d; P < 0.01) than Hep3B cells did. These results implied that the NTS^+^NTR1^+^correlated with the tumor invasion potentials of HCC cell lines.

### NTS/NTR1 co-expression promoted tumor invasion rather than proliferation of HCC cells

Genetically modified HCC cell lines were established to perform the gain of function/loss of function (GOF/LOF) of NTR1 gene. The NTR1-overexpressing HCC cells were constructed by transfecting wild-type Hep3B (Hep3B^wt^) cells with recombinant retroviral vector pLVX-IRES-Puro-NTR1 and hence termed as Hep3B^NTR1hi^ cells. The NTR1-knocked down HCC cells were constructed by silencing Hep3B^wt^ and HepG2^wt^ cells with specific NTR1 siRNAs and thus termed as Hep3B^NTR1si^ and HepG2^NTR1si^ cells (Figure S1a). In RT-qPCR assay and Western blot, the NTR1 expression in Hep3B^NTR1hi^ cells was 17.16 ± 2.17-fold higher than that in Hep3B^wt^ cells (P < 0.01). By contrast, the NTR1 expression in Hep3B^NTR1si^ and HepG2^NTR1si^ cells decreased to (10.33 ± 2.51)% and (11.65 ± 1.62)% of their respective wild-type controls 48 h post-transfection (P < 0.01). However, the secretion of NTS did not change significantly among various genetically modified HCC cells (Figure S1b). This finding showed that stable NTS levels were maintained, although the NTR1 expression was regulated. We then stimulated genetically modified HCC cells with exogenous NTS at a concentration of 1 μg/ml to observe the change in tumor invasion potentials and EMT features and to mimic the strong NTS stimulation in primary HCC tissues.

**Figure 3:**
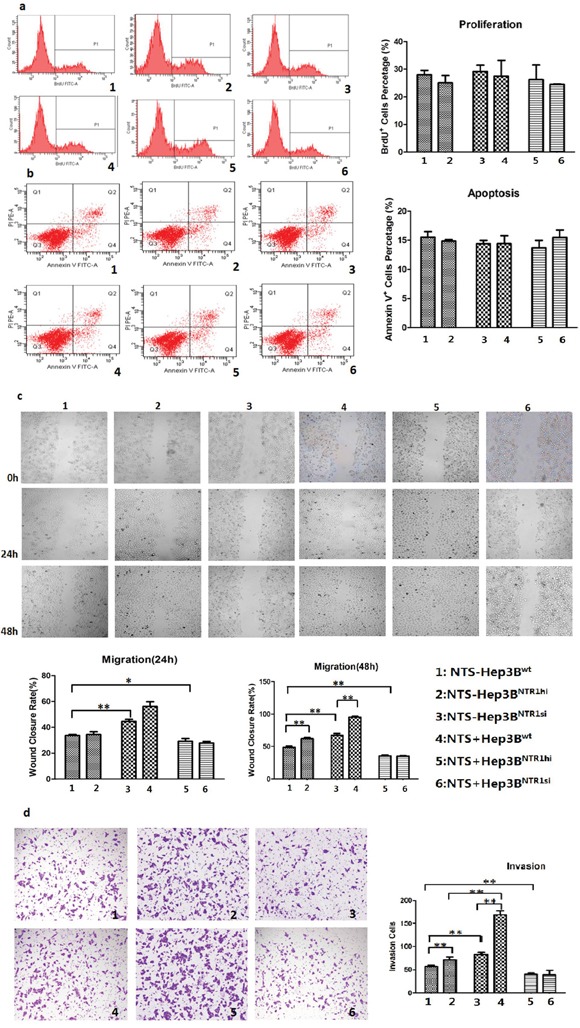
NTS/NTR1 co-expression promoted tumor invasion rather than proliferation of HCC cells **a.** BrdU proliferation assay showed no significant difference was observed among the proliferation rates of Hep3B^wt^, Hep3B^NTR1hi^, and Hep3B^NTR1si^ cells with or without NTS stimulation. **b.** Annexin V apoptosis assay showed no significant difference was detected among the apoptosis rates of Hep3B^wt^, Hep3B^NTR1hi^, and Hep3B^NTR1si^ cells regardless of the presence or absence of exogenous NTS stimulation. **c.** Wound healing test indicated that adding 1 μg/ml of exogenous NTS and/or increasing NTR1 expression promoted the migration capacity of Hep3B cells. **d.** Transwell invasion assay indicated that 1 μg/ml of exogenous NTS and/or increasing NTR1 expression enhanced the invasion potential of Hep3B cells. Note: 1: NTS-untreated Hep3B^wt^ cells; 2: NTS-untreated Hep3B^NTR1hi^ cells; 3: NTS-untreated Hep3B^NTR1si^ cells; 4: NTS-treated Hep3B^wt^ cells; 5: NTS-treated Hep3B^NTR1hi^ cells; 6: NTS-treated Hep3B^NTR1si^ cells.

BrdU proliferation assay was performed to compare the proliferation among various genetically modified HCC cell lines. No significant difference was observed among the proliferation rates of Hep3B^wt^, Hep3B^NTR1hi^, and Hep3B^NTR1si^ cells: 27.93% ± 2.78%, 29.13% ± 4.02%, and 26.20% ± 9.20%, respectively. The proliferation rate did not increase significantly after exogenous NTS stimulation was added (Figure 3a). Annexin V apoptosis assay was conducted to examine the apoptosis among various genetically modified HCC cell lines. No significant difference was detected among the Hep3B^wt^, Hep3B^NTR1hi^, and Hep3B^NTR1si^ cells regardless of the presence or absence of exogenous NTS stimulation (Figure 3b). Similar results were obtained in HepG2^wt^ and HepG2^NTR1si^ cells (Figure S2a/b). These results reveal that the NTS^+^NTR1^+^ did not affect the proliferation and apoptosis of HCC cells.

The migration and invasion of genetically modified HCC cells were evaluated through wound healing test and Transwell invasion assay. The wound closure rates (WCRs) of the Hep3B^NTR1hi^ cell (67.20% ± 4.76%) were significantly higher than those of the Hep3B^wt^ (48.50% ± 3.50%) and Hep3B^NTR1si^ (29.23% ± 3.66%) cells (P < 0.001; Figure 3c). Furthermore, more Hep3B^NTR1hi^ cells migrated across the Matrigel layer than Hep3B^wt^ and Hep3B^NTR1si^ cells, which were 82.67 ± 8.74, 56.67 ± 4.73, and 40.33 ± 4.93 cells/10^4^ cells after 48 h (P < 0.001; Figure 3d). Subsequently, 1 μg/ml of exogenous NTS was added. The 48 h WCRs of the NTS-treated Hep3B^wt^ and Hep3B^NTR1hi^ cells increased significantly compared with that of the NTS-untreated Hep3B^wt^ and Hep3B^NTR1hi^ cells, which were 62.00% ± 2.96% versus 48.50% ± 3.80% (P = 0.008) and 95.50% ± 2.40% versus 67.20% ± 4.76% (P = 0.001), respectively. However, the WCRs did not significantly increase between the NTS-treated Hep3B^NTR1si^ and NTS-untreated Hep3B^NTR1si^ cells (Figure 3c). Consistently, more cells migrated in the NTS-treated Hep3B^wt^ and Hep3B^NTR1hi^ cells than in the NTS-untreated cells, which were 71.67 ± 5.01 cells/10^4^ cells versus 56.67 ± 4.73 cells/10^4^ cells (P = 0.001) and 169.33 ± 14.64 cells/10^4^ cells versus 82.67 ± 8.74 cells/10^4^ cells (P = 0.014), respectively. No significant increase in the migrated cells was observed between the NTS-treated Hep3B^NTR1si^ and NTS-untreated Hep3B^NTR1si^ cells (Figure 3d). Similar results were obtained in HepG2^wt^ and HepG2^NTR1si^ cells (Figure S2c/d). These results implied that the NTS^+^NTR1^+^ significantly promoted tumor migration and invasion rather than stimulated the proliferation and apoptosis of HCC cells.

### NTS/NTR1 co-expression significantly promoted EMT process in HCC cell lines

**Figure 4:**
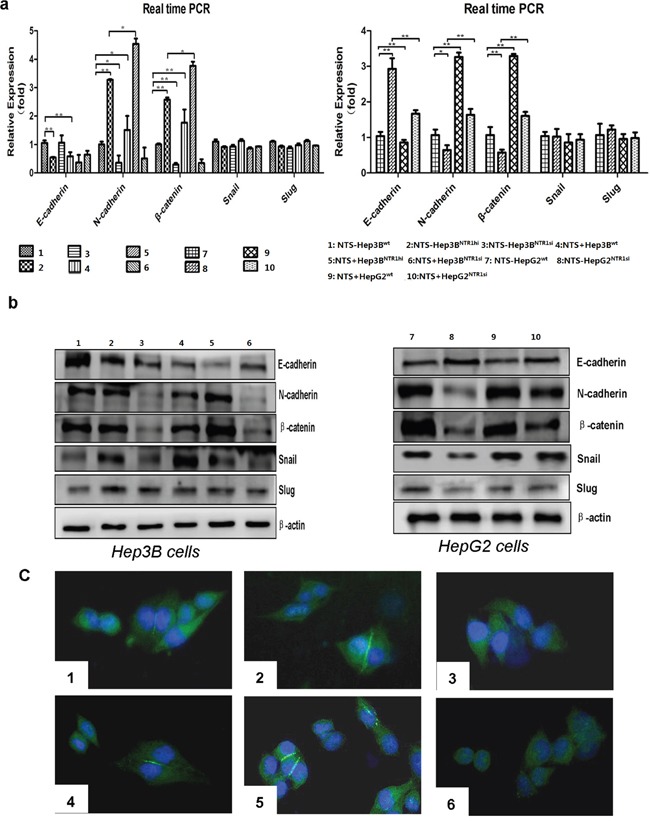
NTS/NTR1 co-expression significantly promoted EMT process in HCC cell lines **a.** The RNA levels of EMT-related markers and transcription factors were examined using real-time RT-PCR. E-cadherin expression in Hep3B^NTR1hi^ cells decreased compared with that in Hep3B^wt^ cells, whereas the expression levels of N-cadherin and β-catenin increased significantly. In Hep3B^NTR1si^ and HepG2^NTR1si^ cells, the expression levels of N-cadherin and β-catenin were lower than those of Hep3B^wt^ and HepG2^wt^ cells. **b.** The protein level of above EMT-related markers and transcription factors were examined using Western blot. N-cadherin and β-catenin proteins significantly increased in Hep3B^NTR1hi^ cells but decreased in Hep3B^NTR1si^ and HepG2^NTR1si^ cells. After 1 μg/ml of exogenous NTS was added, E-cadherin expression decreased and N-cadherin and β-catenin expression increased in Hep3B^wt^, Hep3B^NTR1hi^, and HepG2^wt^ cells. Snail protein increased after the NTR1 expression was enhanced and exogenous NTS stimulation was induced. **c.** To determine NTS-induced alteration of EMT markers in HCC, we examined the distribution of N-cadherin on the cytomembrane through immunofluorescence. N-cadherin increased in the Hep3B^NTR1hi^ cells and exogenous NTS-stimulated Hep3B^wt^ and Hep3B^NTR1hi^ cells but decreased in the Hep3B^NTR1si^ cells regardless of the NTS stimulation. Note: 1: NTS-untreated Hep3B^wt^ cells; 2: NTS-untreated Hep3B^NTR1hi^ cells; 3: NTS-untreated Hep3B^NTR1si^ cells; 4: NTS-treated Hep3B^wt^ cells; 5: NTS-treated Hep3B^NTR1hi^ cells; 6: NTS-treated Hep3B^NTR1si^ cells; 7: NTS-untreated HepG2^wt^ cells; 8: NTS-untreated HepG2^NTR1si^ cells; 9: NTS-treated HepG2^wt^ cells; 10: NTS-treated HepG2^NTR1si^ cells.

We detected the expression of well-known EMT-related protein markers and some transcription factors (TFs), including E-cadherin, N-cadherin, β-catenin, Snail, and Slug, through RT-qPCR assay and Western blot to demonstrate how NTS^+^NTR1^+^ promoted HCC invasion and migration. The results demonstrated that the E-cadherin expression in Hep3B^NTR1hi^ cells decreased compared with that in Hep3B^wt^ cells (P < 0.001), but the expression levels of N-cadherin and β-catenin increased significantly (P < 0.005; Figure 4a). In Hep3B^NTR1si^ and HepG2^NTR1si^ cells, the expression levels of N-cadherin and β-catenin were lower than those of Hep3B^wt^ and HepG2^wt^ cells (P < 0.05; Figure 4a). Consistent results were obtained through Western blot. N-cadherin and β-catenin proteins significantly increased in Hep3B^NTR1hi^ cells but decreased in Hep3B^NTR1si^ and HepG2^NTR1si^ cells (Figure 4b). After 1 μg/ml of exogenous NTS was added, E-cadherin expression decreased and N-cadherin and β-catenin expression increased in Hep3B^wt^, Hep3B^NTR1hi^, and HepG2^wt^ cells (P < 0.05; Figure 4b). Snail protein increased after the NTR1 expression was enhanced and exogenous NTS stimulation was induced. This finding was consistent with the decrease in E-Cadherin in the cytoplasm. We examined the distribution of N-cadherin on the cytomembrane through immunofluorescence (IF) to determine NTS-induced alteration of EMT markers in HCC further. The IF results were consistent with the observations in the Western blot assay, where N-cadherin increased in the Hep3B^NTR1hi^cells and exogenous NTS-stimulated Hep3B^wt^and Hep3B^NTR1hi^cells but decreased in the Hep3B^NTR1si^cells regardless of the NTS stimulation (P < 0.05; Figure 4c). These results suggested that the NTS^+^NTR1^+^stimulated the tumor EMT at RNA and protein levels in HCC.

### NTS-induced EMT correlated with the activation of the canonical Wnt/β-catenin pathway

**Figure 5:**
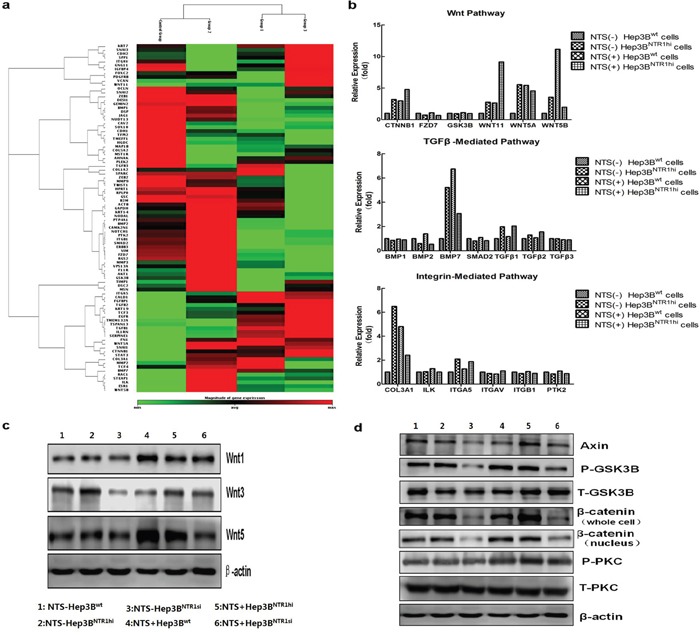
NTS-induced EMT correlated with the activation of the canonical Wnt/β-catenin pathway **a.** A comprehensive pathway analysis focused on 84 EMT-related genes was conducted through quantitative PCR array. The combination of 29 genes displayed a consistent lower expression in Hep3B^wt^ cells but higher expression in Hep3B^NTR1hi^ cells and NTS-treated Hep3B^wt^ cells. **b.** After the pathway was enriched, these 29 genes were distributed on three well-known EMT-related signaling pathways: Wnt, TGF-β, and Integrin pathways. The expression of CTNNB1, WNT5A, WNT 5B, and WNT11 genes dramatically increased in the Hep3B^NTR1hi^ and NTS-treated Hep3B^wt^ cells compared with those in the NTS-untreated Hep3B^wt^ controls. **c.** The protein expression of the Wnt family was examined using Western blot. Wnt1, Wnt3, and Wnt5 were upregulated in Hep3B^NTR1hi^ cells and NTS-treated Hep3B^wt^ cells, but downregulated in Hep3B^NTR1si^ cells. **d.** The expression and phosphorylation levels of some key proteins along the canonical and non-canonical Wnt pathways were detected using Western blot. Axin, p-GSK, and β-catenin proteins along the canonical Wnt pathway were upregulated in the Hep3B^NTR1hi^ and NTS-treated Hep3B^wt^ cells, but were downregulated in the Hep3B^NTR1si^ cells. Note: 1: NTS-untreated Hep3B^wt^ cells; 2: NTS-untreated Hep3B^NTR1hi^ cells; 3: NTS-untreated Hep3B^NTR1si^ cells; 4: NTS-treated Hep3B^wt^ cells; 5: NTS-treated Hep3B^NTR1hi^ cells; 6: NTS-treated Hep3B^NTR1si^ cells.

Various signaling pathways reported to induce EMT in common cancer cells were analyzed to elucidate the molecular mechanisms regulating the NTS-induced EMT in HCC cells. A comprehensive pathway analysis focused on 84 EMT-related genes was conducted through quantitative PCR array. Three different patterns of gene expression profiling were distinguished. The combination of 29 genes displayed a consistent lower expression in Hep3B^wt^ cells but higher expression in Hep3B^NTR1hi^ cells and NTS-treated Hep3B^wt^ cells (Figure 5a). After the pathway was enriched, these 29 genes were distributed on three well-known EMT-related signaling pathways: Wnt, TGF-β, and integrin pathways (Figure 5b). We listed all the genes along these three pathways, compared their expressions in the four groups, and found the most differentially expressed genes were concentrated on the Wnt pathway. After enhancing the NTS signaling by adding exogenous NTS stimulation and/or upregulating the NTR1 expression, the expressions of CTNNB1, WNT5A, WNT 5B, and WNT11 genes dramatically increased to more than two-fold in the Hep3B^NTR1hi^ and NTS-treated Hep3B^wt^ cells compared with those in the NTS-untreated Hep3B^wt^ controls (Figure 5b). Consistently, Hep3B^NTR1si^ cells expressed lower levels of Wnt5a, Wnt5b, and Wnt11 than the Hep3B^wt^ controls (data not shown). These data indicated that the NTS^+^NTR1^+^ stimulated the RNA synthesis of Wnt and β-catenin genes. Similarly, the protein expression of the Wnt family, including Wnt1, Wnt3, and Wnt5, was examined using Western blot (Figure 5c). The results showed that exogenous NTS stimulation and NTR1 expression upregulation promoted the production of all Wnt subtypes. Furthermore, we detected the expression and phosphorylation levels of some key proteins along the canonical and non-canonical Wnt pathways. We confirmed that a series of functional proteins along the canonical Wnt pathway, including Axin, p-GSK, and β-catenin proteins, were upregulated in the Hep3B^NTR1hi^ and NTS-treated Hep3B^wt^ cells but were downregulated in the Hep3B^NTR1si^ cells (Figure 5d). Considering that accumulated β-catenin would translocate to the nucleus, we further analyzed the expression of β-catenin in nuclear lysates. We found a similar increasing pattern of β-catenin in the nucleus of Hep3B^NTR1hi^and Hep3B^wt^cells compared with that in Hep3B^NTR1si^ cells. Nuclear β-catenin protein increased significantly in the NTS-stimulated Hep3B^NTR1hi^ cells (Figure 5d). By contrast, no detectable difference was found in the expression and phosphorylation of the PKC protein, the key regulator along the non-canonical Wnt pathway. These results showed that NTS-induced EMT might be mostly regulated by the activation of the canonical Wnt/β-catenin pathway.

### Inhibition of NTS signaling and Wnt/β-catenin pathway activation reversed the NTS-induced EMT and prevented the tumor invasion of HCC cells

**Figure 6:**
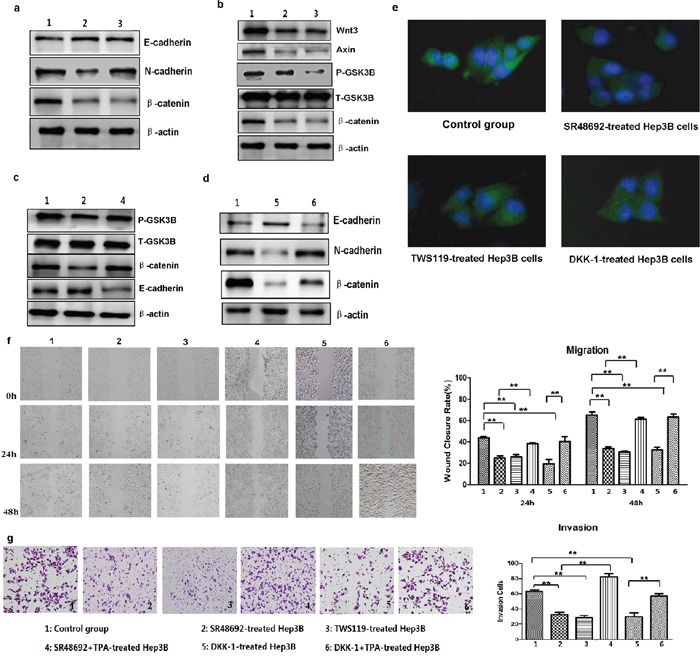
Inhibition of NTS signaling and Wnt/β-catenin pathway activation reversed the NTS-induced EMT and prevented the tumor invasion of HCC cells **a.** Western blot show that blocking NTR1 using SR48692 or inhibiting the Wnt/β-catenin pathway using TWS119 reversed the EMT of Hep3B^wt^ cells. **b.** Western blot revealed that both SR48692 and TWS119 inhibited the activation of the Wnt/β-catenin pathway. **c.** TPA was used to activate GSK3β and the downstream signaling pathway. TPA promoted tumor EMT in SR48692-treated Hep3B^wt^ cells even though SR48692 treatment inactivated the Wnt/β-catenin pathway and inhibited EMT of Hep3B^wt^ cells. **d.** Dickkopf-1 (DKK-1) was used to block the Wnt signaling. DKK-1 reversed the NTS-induced EMT of HCC cells via increasing E-cadherin, decreasing N-cadherin and β-catenin proteins, which was rescued by TPA. **e.** N-cadherin expression in NTS-treated Hep3B^wt^ cells before and after blocking NTR1, GSK3β and Wnt activities was detected by IF, and N-cadherin was significantly downregualted by SR48692, TWS119 and DKK-1. **f.** The migration capacity of NTS-treated Hep3B^wt^ cells after SR48692, TWS119 and DKK-1 treatment was analyzed using wound healing test. The WCR at 24 and 48 h in SR48692, TWS119 and DKK-1-treated Hep3B^wt^ cells decreased dramatically, but TPA almost completely reversed the SR48692-induced and DKK-1-induced inhibition on the migration of NTS-treated Hep3B^wt^ cells. **g.** The invasion potentials of NTS-treated Hep3B^wt^ cells after SR48692, TWS119 and DKK-1 treatment was analyzed using Transwell invasion assay. The cell counts in the Transwell invasion assay declined in SR48692, TWS119 and DKK-1-treated Hep3B^wt^ cells, and TPA fully rescued either the SR48692- or DKK-1-mediated inhibition on tumor invasion in NTS-treated Hep3B^wt^ cells. Note: 1: Control group; 2: SR48692-treated Hep3B cells; 3: TWS119-treated Hep3B cells; 4: SR48692 and TPA-treated Hep3B cells; 5: DKK-1-treated Hep3B cells; 6: DKK-1 and TPA-treated Hep3B cells.

NTR1 antagonist SR48692 and special GSK3β phosphorylation inhibitor TWS119 were used to block the transduction of NTS signaling and the activation of Wnt/β-catenin pathway in NTS-treated Hep3B^wt^ cells and to validate whether NTS induces tumor EMT by activating the canonical Wnt/β-catenin pathway. As predicted, SR48692 significantly reversed the EMT of Hep3B^wt^ cells, which displayed increasing E-cadherin, decreasing N-cadherin, and decreasing β-catenin expressions (Figure 6a). Similarly, TWS119 also reversed the EMT process of Hep3B^wt^ cells (Figure 6a). Therefore, we studied the key functional proteins downstream the canonical Wnt/β-catenin pathway and found that both SR48692 and TWS119 dramatically repressed the expression of Wnt3, Axin, p-GSK3β, and β-catenin in Hep3B^wt^ cells (Figure 6b). TPA was used to activate GSK3β and the downstream signaling pathway in SR48692-treated Hep3B^wt^ cells to determine whether the activation of the Wnt/β-catenin pathway played pivotal roles on NTS-induced EMT. TPA significantly upregulated the phosphorylation level of GSK3β, increased the expression of β-catenin protein, but decreased the expression of E-cadherin protein (Figure 6c). Dickkopf-1 (DKK-1) was used to block the Wnt signaling to confirm the role of the canonical Wnt/β-catenin pathway in NTS-induced EMT further. The results showed that DKK-1 induced similar effects as SR48692 and TWS119 did, which reversed the NTS-induced EMT via increasing E-cadherin, decreasing N-cadherin, and β-catenin proteins (Figure 6d). N-cadherin expression in NTS-treated Hep3B^wt^ cells before and after blocking NTR1, GSK3β, and Wnt activities was detected by IF to confirm the suppressing effects of the inhibitors above. We found that N-cadherin was significantly downregulated by SR48692, TWS119, and DKK-1, which was consistent with the observation in the Western blot assay (Figure 6e). However, attenuated EMT process was rescued by TPA after DKK-1blocking, which significantly enhanced the N-cadherin and β-catenin expressions but inhibited the E-cadherin expression (Figure 6d). These results implied that the activation of the Wnt/β-catenin pathway played a vital role in regulating NTS-induced EMT.

SR48692, TWS119, and DKK-1 significantly inhibited the migration of NTS-treated Hep3B^wt^ cells either at 24 or 48 h. The WCR at 24 h and 48 h in SR48692-treated Hep3B^wt^ cells decreased by nearly two folds compared with that in non-treated Hep3B^wt^ controls, which declined from 43.70% ± 1.37% to 24.86% ± 2.05% at 24 h and from 65.17% ± 2.97% to 33.50% ± 1.70% at 48 h (P < 0.001; Figure 6f). Similarly, the WCR in TWS119-treated and DKK-1-treated Hep3B^wt^ cells declined to 25.90% ± 2.26% and 19.45% ± 4.04% at 24 h, (P < 0.001; P < 0.001) and to 30.29% ± 1.12% and 32.28% ± 2.51% at 48 h, respectively (P < 0.001; P = 0.001; Figure 6f). By contrast, TPA almost completely reversed the SR48692-induced and DKK-1-induced inhibitions on the migration of NTS-treated Hep3B^wt^ cells both at 24 h and 48 h (Figure 6f). Consistently, SR48692, TWS119, and DKK-1 significantly inhibited tumor invasion, in which the cell counts in the Transwell invasion assay declined from 63.33 ± 1.52/10^4^ cells to 32.67 ± 2.51/10^4^ cells, 28.33 ± 1.21/10^4^ cells, and 29.67 ± 5.03/10^4^ cells, respectively (P < 0.001; Figure 6f). However, TPA fully rescued either the SR48692- or DKK-1-mediated inhibition on tumor invasion in NTS-treated Hep3B^wt^ cells (Figure 6g). These results implied that NTS promoted tumor invasion and migration which could be inhibited either by specifically blocking the transduction of NTS signaling or suppressing the activation of the Wnt/β-catenin pathway.

### Blocking NTS signaling inhibited HCC metastasis rather than tumor growth *in vivo*

**Figure 7:**
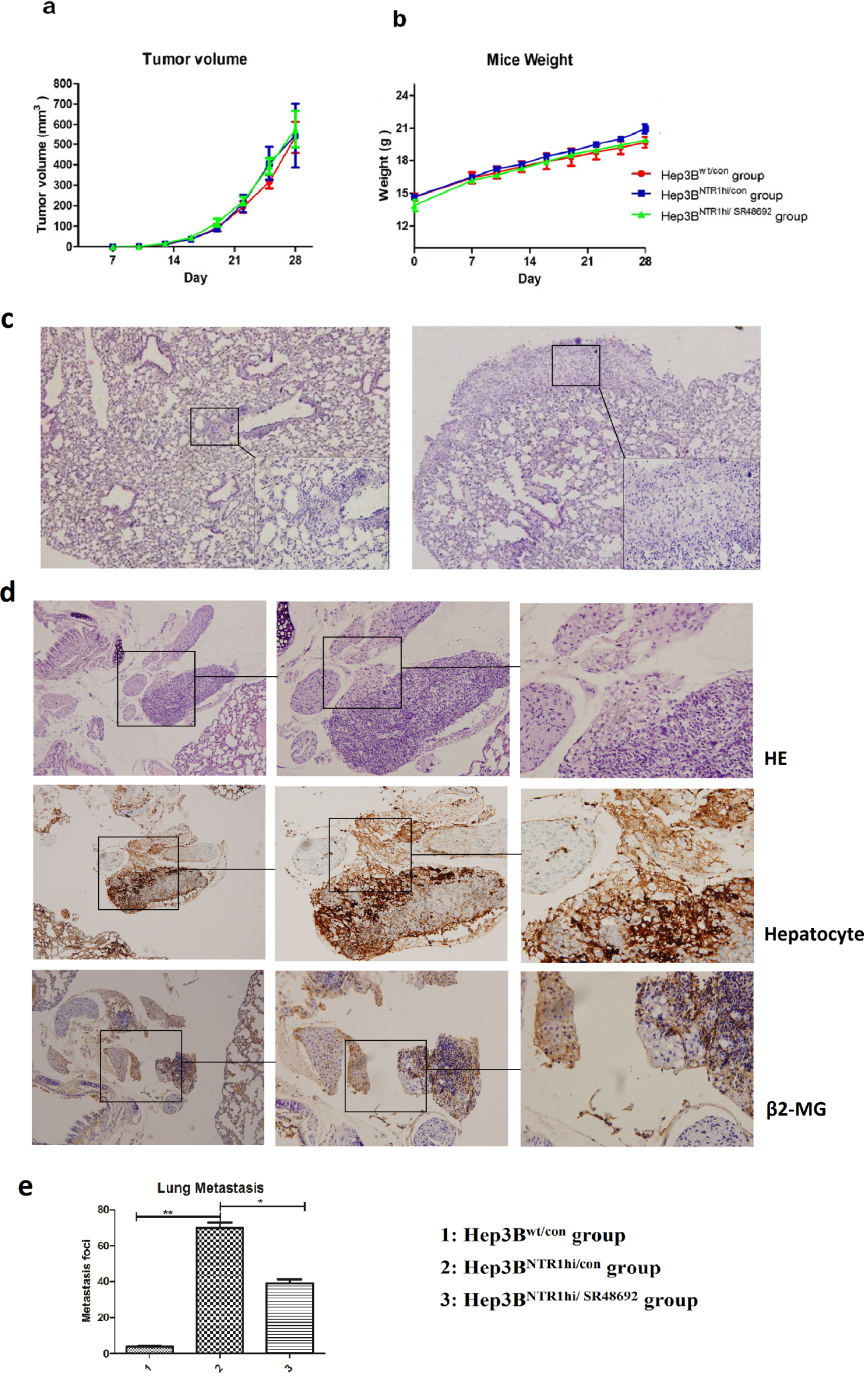
Blocking NTS signaling inhibited HCC metastasis rather than tumor growth *in vivo* **a.** HCC xenograft mouse models were established to study the anti-tumor effects of SR48692 *in vivo*. Hep3B^wt^ and Hep3B^NTR1hi^ cells were subcutaneously implanted in the right armpit, termed as Hep3B^wt/con^ and Hep3B^NTR1hi/con^, respectively. SR48692 diluted in PBS was administrated at 5mg/kg daily for 14 d to block NTS signaling, which was termed as Hep3B^NTR1hi/SR48692^. No significant difference in tumor volumes was observed among Hep3B^wt/con^, Hep3B^NTR1hi/con^, and Hep3B^NTR1hi/SR48692^. **b.** No significant difference in body weights was observed among Hep3B^wt/con^, Hep3B^NTR1hi/con^, and Hep3B^NTR1hi/SR48692^. **c.** Micrometastases in the lungs were detected in Hep3B^wt/con^ and Hep3B^NTR1hi/con^ mice models using HE staining. More lung micrometastases were in Hep3B^NTR1hi/con^ than in Hep3B^wt/con^ mice. **d.** The lung micrometastases were verified using anti-human β2-MG (human derived) and Hepatocyte (liver derived) antibodies through IHC staining. Dual-positive cell mass in mice lungs was regarded as lung micrometastasis. **e.** The numbers of lung micrometastases in subcutaneous Hep3B^NTR1hi/con^ models significantly higher than those in Hep3B^wt/con^ models, and after SR48692 treatment the number of lung micrometastases in Hep3B^NTR1hi/SR48692^ decreased dramatically. Note: 1: Hep3B^wt/con^ group; 2: Hep3B^NTR1hi/con^ group; 3: Hep3B^NTR1hi/SR48692^ group

We established HCC xenograft mouse models to investigate the anti-tumor effects of SR48692 *in vivo*. Hep3B^wt^ and Hep3B^NTR1hi^ cells were subcutaneously implanted in the right armpit and were termed as Hep3B^wt/con^ and Hep3B^NTR1hi/con^, respectively. SR48692 diluted in PBS was administered at 5 mg/kg daily for 14 d to block NTS signaling and termed as Hep3B^NTR1hi/SR48692^. Tumor volumes and body weights didn't significantly differ among Hep3B^wt/con^, Hep3B^NTR1hi/con^, and Hep3B^NTR1hi/SR48692^ (Figures 7a and 7b). Previous studies have reported that lung metastases can be observed in the orthotopic Hep3B tumor models, especially when Hep3B cells were inoculated by intrahepatic implantation. In our pilot study, we found that xenografts of subcutaneous Hep3B^NTR1hi/con^ models displayed more aggressive potentials than Hep3B^wt/con^ models and induced more metastases in lungs at day 45 after the implantation (data not shown). But considering that SR48692 was recommended for treatment less than 14 days in mice, we had to observe the tumor growth and metastases patterns of subcutaneous Hep3B^NTR1hi/con^ models and Hep3B^wt/con^ models for only 2 weeks after the xenografts were detected at day 14. As the literatures reported, characteristic lung metastatic nodules weren't observed in subcutaneous Hep3B models in such a short period of time, but luckily lung micrometastases could be detected under microscopy (Figures 7c). Anti-human β2-MG and Hepatocyte antibodies were used to distinguish minuscule and atypical human liver derived cell mass in mice lungs and dual-positive cell mass was regarded as lung micrometastasis (Figures 7d). We compared the number and size of lung micrometastases among subcutaneous Hep3B^NTR1hi/con^ models, Hep3B^wt/con^ models and Hep3B^NTR1hi/SR48692^ models and found that lung micrometastases in Hep3B^NTR1hi/con^ models were comparably larger and composed of considerable β2-MG and Hepatocyte dual-positive staining cells with distinct HCC morphology. The numbers of lung micrometastases in subcutaneous Hep3B^NTR1hi/con^ models significantly higher than those in Hep3B^wt/con^ models, which was 72.75 ± 11.87 versus 4.18 ± 1.64 (P=0.001; Figures 7e). The number of lung micrometastases in Hep3B^NTR1hi/SR48692^ decreased to 40.67 ± 9.29 after SR48692 treatment was administered (P = 0.012; Figure 7e). These results implied that blocking NTS signaling could inhibit HCC metastasis and NTR1 antagonist SR48692 might be a potential anti-metastasis therapy for NTS^+^NTR1^+^ HCC.

## DISCUSSION

Our previous study on the whole genome expression profiling of Asian HCC cancer samples distinguished a subgroup of HCC characterized by the overexpression of NTS and upregulation of EMT-related genes; in particular, NTS expression is positively correlated with the invasion potential of HCC [14]. However, few reports have focused on the expression and functions of NTS in HCC and the correlation between NTS and tumor EMT. Furthermore, the molecular mechanisms regulating NTS-induced EMT in HCC remain unclear. Therefore, we intended to identify the expression and distribution of NTS and NTR1 and their relationship with EMT in HCC tissues in the present study. In our study we found that NTS was exclusively expressed in tumor tissues and most of the NTS-positive HCC tissues (73.68%) co-expressed NTR1. Previous experiments have identified that NTS could induce the internalization of NTR1 in target cells by a unique recycling cycle. Activated NTR1 traffics from the plasma membrane to early endosomes, and then recycles [23]. Recent study indicated that NTS-induced NTR1 trafficking from plasma membrane to endosomes could be regulated by microRNAs in colonic epithelial cells, thus partially affected NTS/NTR1 complex-activated pro-inflammatory signaling pathways [24]. Similarly, we also found that NTR1 protein expressed both on the cell membrane and cytoplasma in NTS^+^NTR1^+^ HCC samples rather than non-NTS^+^NTR1^+^ samples which implied that the expression and location of NTR1 protein in HCC is also mainly regulated by NTS stimulation. The co-expression of NTS and NTR1 in HCC was correlated with aggressive biological behaviors and poor clinical prognosis. Furthermore, the significant upregulation of MMP9 and multiple EMT markers rather than Ki67 protein in NTS^+^NTR1^+^ HCC tissues implied that the co-expression of NTS and NTR1 promoted HCC invasion and metastasis by inducing EMT in HCC cells. Using a series of genetically modified HCC cell lines, we demonstrated that the increased NTS signaling either by adding exogenous NTS or increasing the NTR1 expression promoted a series of EMT molecular events in HCC cell lines. A specific inhibitor against NTR1 could reverse the NTS-induced EMT and suppress tumor invasion and metastasis both *in vitro* and *in vivo*.

We aimed to explore the molecular mechanism regulating NTS-induced EMT because the NTS signal is a potent trigger of EMT in HCC. PCR array demonstrated that three well-known EMT-related signaling pathways were activated: Wnt, TGF-β, and integrin pathways. We compared the expression levels of all genes in these three pathways among differently NTS-responsible HCC cell lines and found that most differentially expressed genes were concentrated in the Wnt pathway, including CTNNB1, WNT5A, WNT 5B, and WNT11, which remarkably increased by more than twofold after NTS stimulated. Therefore, we focused on the Wnt pathway to elucidate the underlying molecular events regulating NTS-induced EMT. Nineteen known Wnts are present in the mammalian system and are generally divided into two categories, namely, canonical Wnts, including Wnt1, Wnt3A, Wnt8A, and Wnt8B, which activate downstream signaling proteins through the canonical pathway involving β-catenin and non-canonical Wnts, including Wnt4, Wnt5A, and Wnt11 [25]. In the canonical Wnt pathway, the degradation complex consists of adenomatous polyposis coli (APC), Axin, and GSK3β, which induce the phosphorylation of β-catenin and its ubiquitin-dependent degradation in the proteasome. Wnt signals cause the recruitment of dishevelled protein to block GSK3β-mediated phosphorylation of β-catenin and prevent the constitutive destruction of cytosolic β-catenin. In this case, β-catenin accumulates and transports to the nucleus, where it subsequently induces a cellular response by forming a complex with Lefs/TcFs [26]. β-catenin is a proliferation-related transcription factor. However, its effects on tumor invasion and EMT have been gradually recognized [27]. E-cadherin repressor Snail or Slug is also activated by β-catenin–Lefs/Tcfs-mediated transcription [28, 29]. Research on colorectal cancer has demonstrated that β-catenin subsequently increases the expression of EMT-related transcription factors Twist and Snail, along with the upregulation of mesenchymal marker Vimentin and the downregulation of epithelial marker ZO-1 [30]. Non-canonical Wnt pathways, including the planar cell polarity and steroid receptor binding pathways, have been explored. Several studies have shown that the Wnt/Ca^2+^ pathway is associated with cancer cell EMT [31–33], and PKC is a key functional protein regulating the Wnt/Ca^2+^ pathway [34, 35]. Although an increase in Wnt5 expression was observed through PCR array and Western blot, the non-canonical Wnt/Ca^2+^ pathway was not considered as the vital signaling pathway regulating the NTS-mediated EMT in HCC because no detectable evidences of the PKC protein activation were observed after NTS stimulation was induced or NTR1 expression was increased. By contrast, most of the functional signaling proteins downstream the canonical Wnt pathway were upregulated after NTS stimulation was added or NTR1 expression was enhanced, including Axin, p-GSK, and β-catenin, either by PCR array or Western blot. Blocking experiments indicated that the NTR1 antagonist SR48692, Wnt inhibitor DKK-1, and GSK-3β inhibitor TWS119 could block the canonical Wnt/β-catenin pathway and inhibit NTS-induced EMT with a similar efficiency. Furthermore, the repressive effects of SR48692 on NTS-induced EMT and tumor invasion were rescued by specific GSK-3β antagonist TPA in HCC cells. SR48692 inhibited the lung metastases of NTR1-overexpressing HCC xenografts *in vivo* in tumor-bearing mouse models. Thus, we proposed that the NTS^+^NTR1^+^ induced EMT by activating the canonical Wnt/β-catenin pathway.

**Figure 8:**
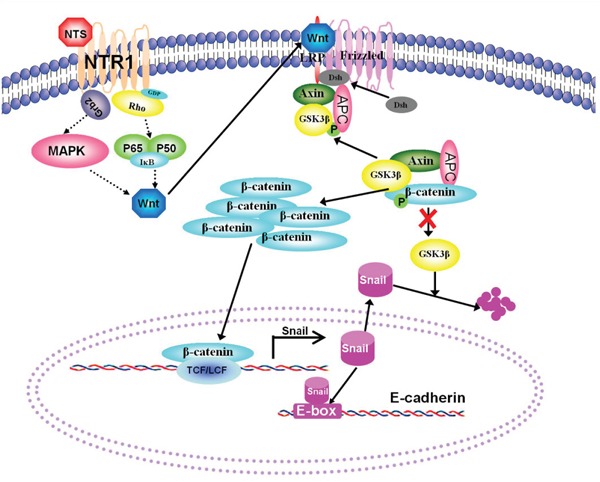
A hypothesis of the “NTS/NTR1-Wnt-EMT” axis was proposed to describe NTS/NTR1-induced EMT in HCC cells A diagram was generated to display the speculated signaling network downstream NTR1 and the interaction among a series of molecules regulating the activation of the Wnt/β-catenin pathway. The interaction of NTS and NTR1 induces the activation of Ras-dependent mitogen-activated protein kinase (MAPK) downstream cascades and the NF-κB pathway which promotes the production of Wnt proteins. Enhanced Wnt signaling causes the recruitment of dishevelled protein to block GSK3β-mediated phosphorylation of β-catenin, whose ubiquitin-dependent degradation in the proteasome increases β-catenin accumulates and transports to the nucleus, subsequently induces cellular responses via forming a complex with Lefs/TcFs. The transcription of Snail is upregulated by β-catenin-Lefs/Tcfs complex and the degradation of Snail is suppressed by attenuated GSK3β activity, which increased the expression of Snail in plasma and then inhibits E-cadherin expression by binding to E-box in the promoter of E-cadherin gene. Therefore, HCC cells displayed characteristic EMT features, including decreasing expression of E-cadherin and accumulation of β-catenin in cytoplasm.

We presented a diagram (Figure 8) to display the speculated signaling network downstream NTR1 and the interaction among a series of molecules after NTS interacted with NTR1, which may elucidate how NTS signaling induced the activation of the Wnt/β-catenin pathway and thus promoted tumor EMT in HCC. The diagram could clearly demonstrate our hypothesis of the “NTS/NTR1-Wnt-EMT”axis in HCC, which included a series of complex molecular events during NTS-induced EMT of HCC cells and tumor invasion *in vitro* and *in vivo*. Studies on colorectal carcinoma have indicated some key NTR1 downstream signaling pathways regulating Wnt expression [36]. The interaction of NTS and NTR1 induces the activation of Ras-dependent mitogen-activated protein kinase (MAPK) downstream cascades and the NF-κB pathway [37]. The p38 MAPK is necessary to the Wnt3-induced accumulation of β-catenin and Lef/Tcf-sensitive gene activation, and the specific inhibition of p38 MAPK restores the Wnt3a-attenuated GSK3β kinase activity [38]. Wnt5a transcription is regulated by NF-κB; in this process, a conserved NF-κB-binding site within the Wnt5a promoter participates in the tumor necrosis factor-alpha (TNFα) and the Toll-like receptor (TLR) induces Wnt5a upregulation in inflammation [39]. However, further studies are needed to elucidate whether other signaling pathways are involved in the NTS-induced Wnt expression, which might provide new insights into the screening of novel anti-cancer targets in HCC.

This study is the first to demonstrate that the co-expression of NTS and NTR1 in HCC is correlated with tumor invasion and poor prognosis. Thus, their co-expression can be regarded as a predictive biomarker of aggressive phenotype and an independent prognostic factor of poor clinical outcome in HCC. We also elucidated the relationship between the NTS signaling and tumor EMT at cellular and tissue levels. We further demonstrated that blocking the NTS signaling by disrupting the interaction between NTS and NTR1 could significantly inhibit EMT and tumor invasion *in vitro* and *in vivo*. We also identified that the dysfunctional activation of the Wnt/β-catenin pathway regulating the NTS-induced EMT and tumor invasion in HCC which could be suppressed significantly both *in vitro* and *in vivo* through target therapy against NTS signaling, such as the specific antagonist against NTR1 SR48692. SR48692 has elicited potent anti-tumor efficiency in the treatment of advanced colorectal cancer and small cell lung cancer since the early 2000s by inhibiting NTS-induced tumor proliferation [40]. Studies on prostate carcinoma have also validated the clinical safety and anti-tumor effects of SR48692 [41]. In our study, SR48692 could significantly inhibit the metastases of NTR1-overexpressing HCC xenografts in the lungs *in vivo*. Therefore, we proposed a hypothesis that the “NTS/NTR1-Wnt-EMT” axis existed and promoted tumor invasion of HCC cells. This hypothesis may represent an underlying pro-metastatic molecular mechanism inducing early relapse after surgery and poor clinical outcome in HCC patients. Furthermore, target therapy against NTS signaling may be a new promising anticancer therapeutic strategy to prevent tumor metastasis and to improve the clinical prognosis of HCC patients.

## MATERIALS AND METHODS

### Patient information

This study recruited 100 cases of HCC patients treated with partial liver resection surgery at the Department of Hepatobiliary Oncology of the Tianjin Medical University Cancer Institute and Hospital from November 2007 to November 2009. These patients included 93 males and 7 females with a median age of 54.5 years. The patients were pathologically diagnosed with HCC at histological grade I (*n* = 25), grade II (*n* = 54), and grade III (*n* = 21), and classified as stage I (*n* = 26), stage II (*n* = 36), and stage III (*n* =38; Table 1). No prior treatments, including chemotherapy or radiotherapy, were conducted before liver resection surgery was performed. Postoperative follow-up time was 40–65 months. This project was approved by the Ethics Committee of Tianjin Medical University. All experiments were performed in accordance with the principles of the Declaration of Helsinki. Written consents were obtained from the patients.

### Cell lines and cell culture

Human HCC cell lines 7721, MHCC97L, Hep3B, and HepG2, normal liver cell line L02, and human embryonic kidney cell line 293T were obtained from the Chinese Academy of Medical Sciences. Lentivirus pLVX-IRES-Puro plasmids and packaging plasmids (pSPAX2 and pMD2G) were purchased from Clontech (Mountain View, CA, USA). *Eshcerichia coli* Dh5α competent cells were purchased from Invitrogen (Grand Island, NY, USA). The cell lines were maintained in 10% fetal bovine serum (FBS)/Dulbecco's modified Eagle's medium (DMEM; Gibco BRL, Grand Island, NY, USA) in a humidified chamber with 5% CO_2_ at 37 °C. NTS (Phoenix Pharmaceuticals, NY, USA) was dissolved in PBS as a 1 mg/ml stock and stored at −80 °C. SR48692 (Sigma, St. Louis, MO, USA) was also dissolved in dimethyl sulfoxide (DMSO) as a 2 mM stock and stored at −80 °C. TWS119 (Millipore, Billerica, MA, USA) and TPA (Cell Signaling Technology, Danvers, MA, USA) were dissolved in DMSO as a 1 mM stock and stored at −80 °C. DKK-1 (Peprotech, Offenbach, USA) was dissolved in PBS as a 10 μg/ml stock and stored at −80 °C. HCC cells were incubated with 1 μg/ml NTS, 10 nM SR48692, 5 nM TWS119, 200 nM TPA, and 200ng/ml DKK-1 at 37 °C for 24 h.

### Construction of genetically modified HCC cell lines

Total cell RNA was isolated from HepG2 cells by using a thiocyanate reagent (Trizol; Invitrogen, Grand Island, NY, USA). RNA (2 μg) was reverse-transcribed with Moloney murine leukemia virus-reverse transcriptase (M-MLV; Takara, Tokyo, Japan) in accordance with the manufacturer's instructions. RT mix was then used to amplify the full-length cDNA of human NTR1 through PCR by using *Taq* DNA polymerase (Takara, Tokyo, Japan) under the following conditions: 95 °C for 5 min; 30 cycles at 95 °C for 1 min, 58 °C for 1 min, and 72 °C for 1 min; and 72 °C for 10 min. The forward primer containing an EcoRI site was 5′-TgaattcATGCGCCTCAACAGCTCCGCGCCGG-3′, and the reverse primer containing a Xbal site was 5′-TATAtctagaCTAGTACAGCGTCTC-3′. Both the 1256 bp NTR1 fragment and the puromycin-resistant retroviral vector pLVX-IRES-Puro were digested with EcoRI and Xbal (Takara, Tokyo, Japan). The NTR1 fragment was ligated into the digested pLVX-IRES-Puro by using T4 DNA ligase (Toyobo, Tokyo, Japan), and the identity of the recombinant plasmids was confirmed through DNA sequencing. The constructed vector was named pLVX-IRES-Puro-NTR1. The pLVX-IRES-Puro-NTR1, pSPAX2, and pMD2G were mixed at 1:1:1, which was used to transfect 293T mixtures with Lipofectamine 2000 (Invitrogen, Grand Island, NY, USA) in accordance with the manufacturer's instructions. Supernatants were collected 48 h after transfection and filtered through 0.45 μm disc filters, which were either used immediately or stored at −80 °C. Hep3B cells were seeded in 6-well cell culture clusters and incubated with the medium containing 1 volume of filtered virus-containing supernatant and 9 volumes of fresh growth media (MOI = 30) for 24 h in the presence of 8 μg/ml Polybrene (Sigma, St. Louis, MO, USA). After 48 h, the infected cells were incubated in the media containing 10% FBS and 2 μg/ml of puromycin. The selected positive cells were pooled, named Hep3B^NTR1hi^, and used for further experiments.

Hep3B and HepG2 cells seeded in 6-well cell culture clusters were transfected with siRNA smart pool targeting the mRNA of human NTR1 by using Lipofectamine 2000 (Invitrogen, Grand Island, NY, USA). The medium was discarded, and 500 μl of transfection mixture containing 5 μl of Lipofectamine 2000 and 10 μl of 20 μM NTR1 siRNA (GenePharma, Shanghai, China) in Opti-MEM were added to obtain a final concentration of 100 nM siRNA. Control cells were transfected with the same amount of non-targeting siRNA. Transfected cells were cultured for 72 h and harvested for RT-PCR validation. The transfected cells were named Hep3B^NTR1si^or HepG2^NTR1si^.

### Immunohistochemistry

All of the 100 cases of paraffin-embedded HCC samples were collected from the Department of Pathology of the Tianjin Cancer Institute and Hospital. The samples were heated for 1 h at 60 °C, deparaffinized in xylene, and rehydrated with graded alcohol. Antigens were retrieved by heating the samples in citrate buffer (pH 6.0) for 2 min. Endogenous peroxidase activity was quenched in a methanol and hydrogen peroxide bath for 20 min. All of the samples were incubated overnight at 4 °C with mouse anti-human NTS, NTR1, Ki67, MMP9, E-cadherin, N-cadherin, β-catenin, and Vimentin monoclonal antibodies (Santa Cruz Biotech, Dallas, TX, USA) at dilution of 1:500, and a biotinylated secondary antibody goat anti-mouse IgG (Santa Cruz Biotech, Dallas, TX, USA) labeled with streptavidin-horseradish peroxidase (HRP) by using a DAB staining kit (Maixin Biotechnology, Fuzhou, China). For the negative control, the primary antibody was substituted with mouse isotype IgG1. Positive cells were stained brownish yellow in the cytoplasm or in the cell membrane. Images were obtained by using an Olympus BX51 microscope. Five representative high-power fields (400× magnification) were selected for each tissue section for histological evaluation. For each protein, two parameters, namely, positive rate (PR) and staining intensity (SI), were used to describe the expression on the basis of the extensity and intensity of the positively stained cells in the samples. PR denotes the percentage of positively stained cells in cancer tissues: ≤10%, negative (scored as 0); 11%–30%, positive at low frequency (scored as 1); 31%–60%, positive at medium frequency (scored as 2); and ≥60%, positive at high frequency (scored as 3). SI refers to the ranked staining intensity of positively stained cells in HCC samples. This value ranged from 0 to 3, which corresponded to negative, weakly positive, moderately positive, and strongly positive. The sum represented the final scores of each protein marker in the HCC samples because protein expression was comprehensively evaluated on the basis of both parameters. A final score of <4 was defined as low/negative expression and a final score of ≥4 was defined as high expression.

### Cell proliferation assay

The proliferation of Hep3B^wt^, Hep3B^NTR1hi^, Hep3B^NTR1si^, HepG2^wt^, and HepG2^NTR1si^ cells was compared through BrdU cell proliferation assay (BD Biosciences, San Jose, CA, USA) in accordance with the manufacturer's instructions. In brief, the control and transfected cells were seeded at a density of 5 × 10^4^ cells/well in 24-well cell culture clusters and cultured for 48 h. BrdU dilution (10ng/ml) was added to the culture media 12 h before the proliferation assay was completed. The HCC cells were harvested, resuspended in 200 μl of washing buffer, and incubated with FITC-labeled anti-BrdU antibody. Incorporation was detected through flow cytometry to measure the percentage of BrdU-positive cells.

### Cell apoptosis assay

The apoptotic ratios of cells were determined using an Annexin V-FITC apoptosis detection kit (BD Biosciences, San Jose, CA, USA). After 12 h of starvation treatment, the cells were collected and washed twice with cold PBS buffer, resuspended in 100 μl of binding buffer, incubated with 5 μl of FITC-labeled Annexin V and 10 μl of PI for 15 min at room temperature, and analyzed through flow cytometry. The DMSO-treated cells were used as negative control.

### Scratch wound healing assay

The cells were cultured in a serum-free medium for 12 h to confluency, and scratched with a sterile pipette tip. The cells were washed twice with PBS to remove cell debris. The culture medium was replaced with a growth medium containing 5% FBS for 48 h. Wound areas were photographed and analyzed using IPP 6.0 at 100× magnification. Each wound was evaluated six times, and the average was obtained. Independent duplicates were examined in the same manner. Wound closure rate (WCR) was measured photogrammetrically at 24 and 48 h in accordance with the following equation: WCR = {1 − [(wound area)/(original wound area)]}.

### Invasion assay

Invasion assay was performed with 24-Transwell chambers (BD Biosciences, San Jose, CA, USA). In brief, 1 × 10^4^ cells were harvested and resuspended in 200 μl of DMEM without serum and then plated in the top chamber. The lower chamber of the Transwell was filled with 500 μl of DMEM supplemented with 10% FBS. A cell suspension was applied onto the Matrigel membrane and incubated at 37 °C for 24 h. The cells were passed through the Matrigel, and the filtered cells were fixed with 70% methanol, stained with 0.2% crystal violet, washed with PBS, and counted under a microscope.

### Real-time PCR Analysis

Total RNA was isolated through Trizol extraction in accordance with the manufacturer's instructions and reverse-transcribed with M-MLV (Takara, Tokyo, Japan). cDNA was amplified using SYBR *Premix Ex Taq*™ (Takara, Tokyo, Japan) in a 7500 RT-PCR System (Applied Biosystems, Foster City, CA, USA). Comparative RT-PCR was performed in triplicate, and the relative gene expression was calculated by employing the ΔΔ cross threshold (Ct) method. The following primers were used: NTS: FP, 5′-GAACAGCCCAGCTGAGGAAA-3′; RP, 5′-TCCTGGATTAACTCCCAGTGT-3′;E-cadherin:FP, 5′- TGAAGGTGACAGAGCCTCTGGAT-3′;RP, 5′-TGGG TGAATTCGGGCTTGTT-3′;β-catinin: FP, 5′-TGGTG ACAGGGAAGACATCA-3′; RP, 5′-CCATAGTGAAGG CGAACTGC-3′;N-cadherin:FP, 5′-ACAGTGGCCACC TACAAAGG-3′; RP, 5′-CCGAGATGGGGTTGATAA TG-3′; Snail: FP, 5′-AGCCTGGGTGCCCTCAAGAT-3′; RP, 5′-AGGTTGGAGCGGTCAGCGAA-3′; and Slug: FP, 5′-TGCCTGTCATACCACAACCAGA-3′; RP, 5′-GG AGGAGGTGTCAGATGGAGGA-3′.

### Western blot analysis

The cells were harvested and washed twice with PBS (pH 7.4, 0.15 M). Total protein was extracted by RIPA buffer (Beyotime, Shanghai, China). Approximately 30 μg of total protein was subjected to SDS-PAGE and transferred to PVDF membranes. The membranes were blocked with 5% skim milk in TBST and incubated with the primary antibody (1:1000) in TBST containing 5% BSA overnight at 4 °C. The membranes were washed twice with TBST and incubated with HRP-conjugated secondary antibody (1:4000; Santa Cruz Biotech, USA) at room temperature for 2 h. The membrane was exposed using an enhanced chemiluminescence reagent (Chemicon International, USA). The membranes were then reprobed with anti-β-actin antibody (Santa Cruz Biotech, Dallas, TX, USA) in 1:2000 dilutions to confirm equal protein loading.

### Immunofluorescence

The cells were plated in 12-well glass chamber slides, and cell growth was arrested after 24 h. In brief, the cells were fixed with 4% paraformaldyhyde and blocked. The slides were treated with 0.05% Triton and then incubated with rabbit anti-N-cadherin (1:100) antibody overnight at 4 °C. N-cadherin staining was completed with FITC-conjugated goat anti-rabbit IgG1 (1:400) at RT in the dark for 1 h and then detected under a fluorescence microscope.

### PCR array

Differentially expressed genes were compared before and after NTS stimulation by using a human EMT RT^2^ profiler PCR array kit (QIAGEN, Hilden, Germany). PCR was performed on a 7500 RT-PCR system (Applied Biosystems, Foster City, CA, USA) in accordance with the manufacturer's instructions. For data analysis, ΔΔ Ct method was performed and analyzed using specific algorithms provided by the manufacturer. Fold-changes were then calculated using QiaGen (www.SABiosciences.com/pcrarraydataanalysis.php).

### Animal model

Hep3B cells were used to establish an orthotopic murine HCC model and to examine the anti-tumor efficiency of different drugs and treatments, such as gemcitabine, sorafenib, and microsecond pulsed electric fields. Hep3B xenograft models have also been developed to investigate HCC metastasis *in vivo* [42, 43]. NOD-SCID BALB/c mice were inoculated subcutaneously in the right armpit with 2 × 10^6^ Hep3B^wt^ or Hep3B^NTR1hi^ cells; the growth of the primary tumors and the number of lung metastases were compared. SR48692, which is the specific antagonist of NTR1, was dissolved and stocked with DMSO at a concentration of 2 mM. SR48692 was diluted in PBS and inoculated (100 μl) i.p. at a concentration of 5 mg/kg in mice daily for 14 days to block NTS signaling *in vivo*. The control mice were treated with blank PBS containing 10% (v/v) DMSO. Tumor growth was evaluated through caliper measurements, and tumor volume was calculated in accordance with the following ellipsoid formula: volume = tumor length ×width^2^ × 0.5. The mice were weighed weekly after tumor was implanted. The mice were sacrificed 4 weeks after the tumor was implanted. Their lungs were resected and embedded in paraffin, and the micrometstases in their lungs were counted under a microscope. In order to define the lung micrometstases were of human live origin, two anti-human β2-MG(Cell Signaling Technology, USA) and Hepatocyte (Zhong Shan-Golden Bridge, Beijing, China) antibodies were used to distinguish minuscule and atypical cell mass in mice lungs. Two experienced pathologists reviewed all IHC slides of lung samples and evaluated the numbers and sizes of lung micrometastases. The experiment was approved by the Ethics Committee for Animal Experiments of the Tianjin Medical University Cancer Hospital and Institute and was performed in accordance with the Guide for the Care and Use of Laboratory Animals.

### Statistical analysis

Data were analyzed using SPSS 17.0. Measurement data were presented as median (interquartile range) and compared through χ^2^ test. Quantitative data were presented as mean ± standard deviation and compared through ANOVA and LSD tests. Spearman's rank order test and linear regression analysis were performed to assess the correlations between protein levels. Cumulative survival was determined via Kaplan–Meier method. Univariate survival analysis between the different protein markers and the overall survival of HCC patients was conducted through log-rank test. Multivariate survival analysis was performed using a Cox proportional hazard model. Independent prognostic factors were screened using a forward stepwise procedure. Statistical significance was set at P <0.05.

## SUPPLEMENTARY FIGURES


