# Supplementary material for: NTS/NTR1 co-expression enhances epithelial-to-mesenchymal transition and promotes tumor metastasis by activating the Wnt/β-catenin signaling pathway in hepatocellular carcinoma
Source: Oncotarget. 2016 Sep 6;7(43):70303–22. doi: 10.18632/oncotarget.11854 (PMC5342554; doi:10.18632/oncotarget.11854)
Supplement: Supplementary file 1 [file oncotarget-07-70303-s001.pdf]

NTS/NTR1 co-expression enhances epithelial-to-mesenchymal transition and promotes tumor metastasis by activating the Wnt/β-catenin signaling pathway in hepatocellular carcinoma

SUPPLEMENTARY FIGURES

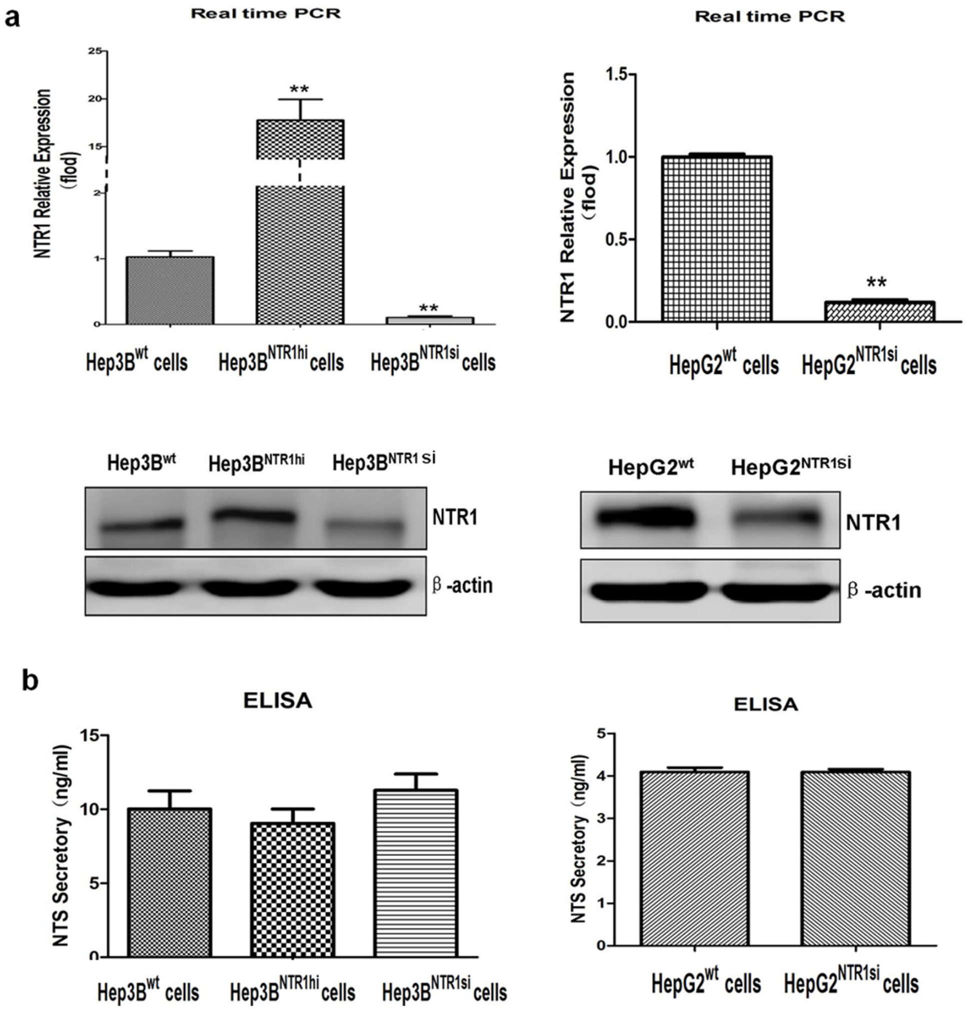

Supplementary Figure S1: Construction and verification of NTR1-genetically modified HCC cell lines. a. $Hep3B^{NTR1hi}$ cells were constructed by transfecting $Hep3B^{wt}$ cells with recombinant retroviral vector pLVX-IRES-Puro-NTR1, and $Hep3B^{NTR1si}$ and $HepG2^{NTR1si}$ cells were constructed by silencing $Hep3B^{wt}$ and $HepG2^{wt}$ cells with specific NTR1 siRNAs. The NTR1 expression in different HCC cells was detected using qRT-PCR and Western blot. $Hep3B^{NTR1hi}$ cells expressed higher level of NTR1, whereas $Hep3B^{NTR1si}$ cells expressed lower level of NTR1 comparing with $Hep3B^{wt}$ cells. Similarly, compared to $HepG2^{wt}$ cells, $HepG2^{NTR1si}$ cells expressed lower level of NTR1 dramatically. **b.** The secretion of NTS in different HCC cells was detected using ELISA assay. No significant difference of the secretion of NTS was observed among various genetically modified HCC cells.

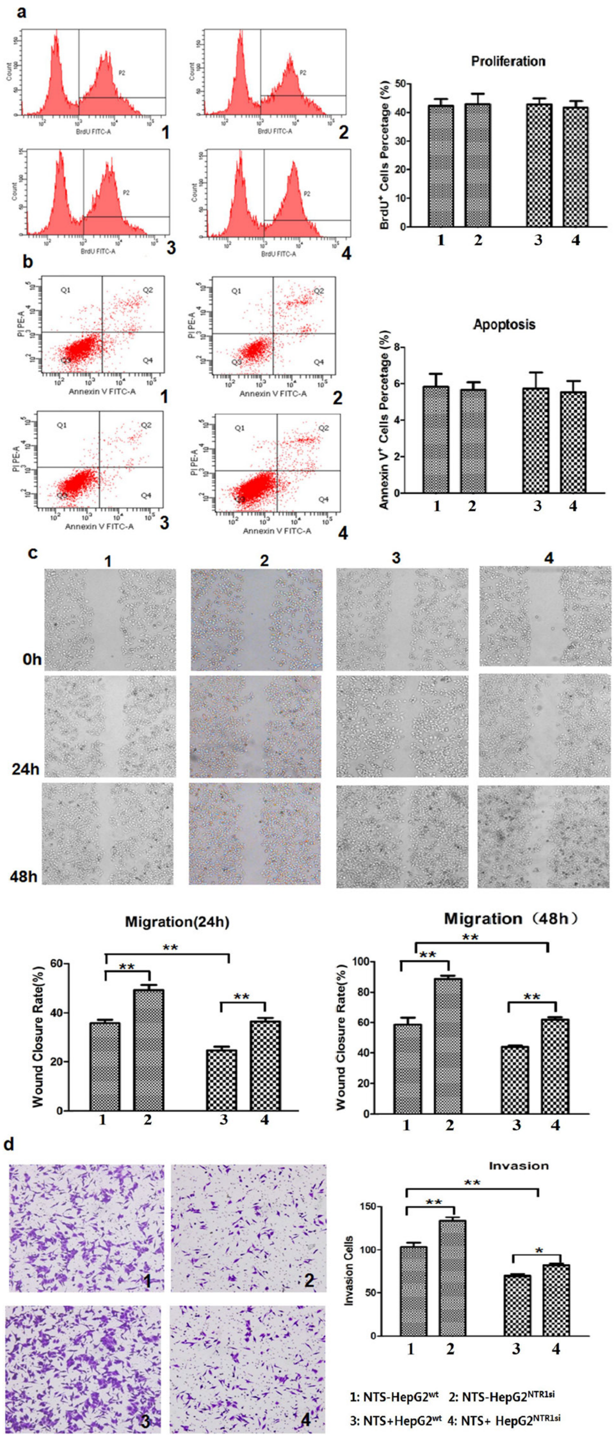

Supplementary Figure S2: NTS/ NTR1 co-expression promoted tumor invasion rather than proliferation of HepG2 cells. a. BrdU proliferation assay showed no significant difference was observed between the proliferation rates of HepG2^{wt} and HepG2^{NTR1si} cells with or without NTS stimulation. **b.** Annexin V apoptosis assay showed no significant difference was detected between the apoptosis rates of HepG2^{wt} and HepG2^{NTR1si} cells regardless of the presence or absence of exogenous NTS stimulation. **c.** Wound healing test indicated that adding 1 μg/ml of exogenous NTS and/or increasing NTR1 expression promoted the migration capacity of HepG2 cells cells. **d.** Transwell invasion assay indicated that 1 μg/ml of exogenous NTS and/or increasing NTR1 expression enhanced the invasion potential of HepG2 cells. Note: 1: NTS-untreated HepG2^{wt} cells; 2: NTS-untreated HepG2^{NTR1si} cells; 3: NTS-treated HepG2^{wt} cells; 4: NTS-treated HepG2^{NTR1si} cells.
